# Editorial Note: Discovery of Markers of Exposure Specific to Bites of *Lutzomyia longipalpis*, the Vector of *Leishmania infantum chagasi* in Latin America

**DOI:** 10.1371/journal.pntd.0012519

**Published:** 2024-09-20

**Authors:** The PLOS Neglected Tropical Diseases Editors

Following the publication of this article [1], concerns were raised regarding results presented in Figs 1 and 5. Specifically, when levels are adjusted to visualize the background:

- In Fig 1B, there appears to be a vertical discontinuity in background between the “-” and “+” lanes in the Dog panel.
- In Fig 5, there does not appear to be signal in lane 7 of the LJL13 Human panel or in lane 6 of the LJL23 Dog panel. There also appear to be vertical discontinuities in the background on either side of these lanes.

The corresponding author provided additional methodological information to explain the appearance of vertical discontinuities:

Western blots were performed using the mini-protean II multiscreen apparatus (Bio-Rad, Hercules, CA). This technique results in spaces appearing between the raised lanes where there is no contact with the primary antibody, and when the blots are scanned into a computer, the raised effect of each lane is lost and each lane appears as a separate blot (see example images in S1 and S2 Files). In the LJL23 Dog panel in Fig 5, there was no reaction to the LJL23 antigen in the negative control sample in lane 6 and in the LJL13 Human panel of Fig 5, lane 7 had no sample and was used to separate samples on the left from samples on the right.

The corresponding author provided the available uncropped underlying images for this article (S3–S7 Files) including the Dog and Fox panels of Fig 1B, and the Dog LJL23 panel of Fig 5, but they were unable to retrieve all original images for the figures included in the article.

In evaluating the “-” lane of the Dog panel of Fig 1B, PLOS noted some differences between the published figure and the underlying blot image. Although this issue was not resolved, PLOS concluded that the data provided for Figs 1B and 5 appear to support the results reported in [1].

## Supporting information

S1 File Example of a photograph of an underlying image showing a multiscreen apparatus blot. This is an image of a multiscreen apparatus blot from an experiment unrelated to this article, provided to show the discontinuity in background seen in blots used in this methodology.
(JPG)

S2 File Example of a scanned underlying image showing a multiscreen apparatus blot. This is a scanned image of a multiscreen apparatus blot from an experiment unrelated to this article, provided to show the discontinuity in background seen in blots used in this methodology. Once scanned, the image appears flat and the negative lane appears similar to the blank background.
(JPG)

S3 File Original, uncropped image underlying the Dog and Fox panels of Fig 1B. (JPG)

S4 File Original, uncropped image underlying the left panel of Fig 2B. (JPG)

S5 File Original, uncropped image underlying the LJL23 Dog panel of Fig 5. (JPG)

S6 File Original, uncropped image underling the LJM11 Human VL endemic area panel of Fig 6. (JPG)

S7 File Original, uncropped image underling the LJM17 Human VL endemic area panel of Fig 6. (JPG)
