# Supplementary figures and images for: Editorial Note: Discovery of Markers of Exposure Specific to Bites of Lutzomyia longipalpis, the Vector of Leishmania infantum chagasi in Latin America
Source: PLoS Negl Trop Dis. 2024 Sep 20;18(9):e0012519. doi: 10.1371/journal.pntd.0012519 (PMC11415079; doi:10.1371/journal.pntd.0012519)

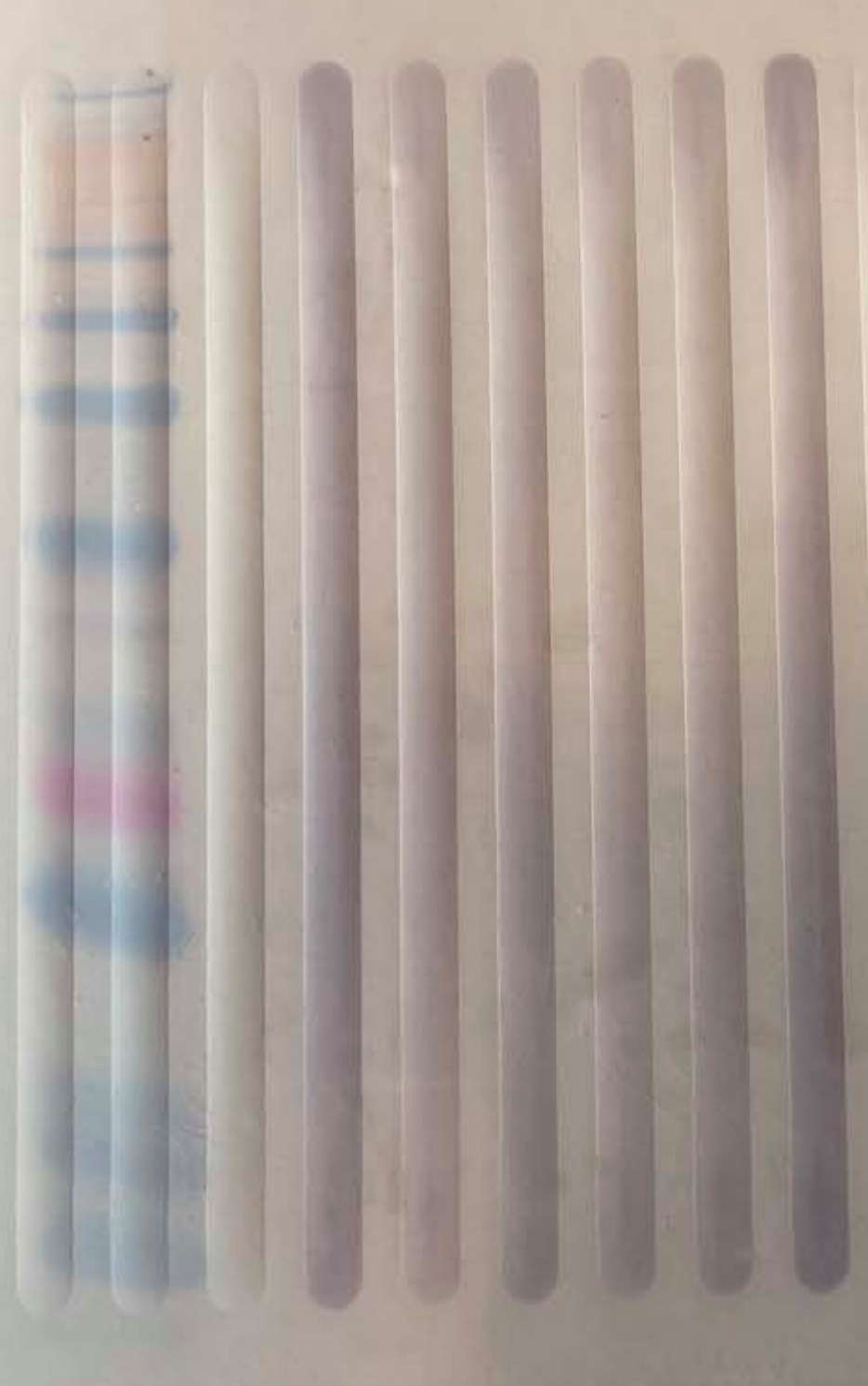

Supplement: S1 File — This is an image of a multiscreen apparatus blot from an experiment unrelated to this article, provided to show the discontinuity in background seen in blots used in this methodology. (JPG) [file pntd.0012519.s001.jpg]

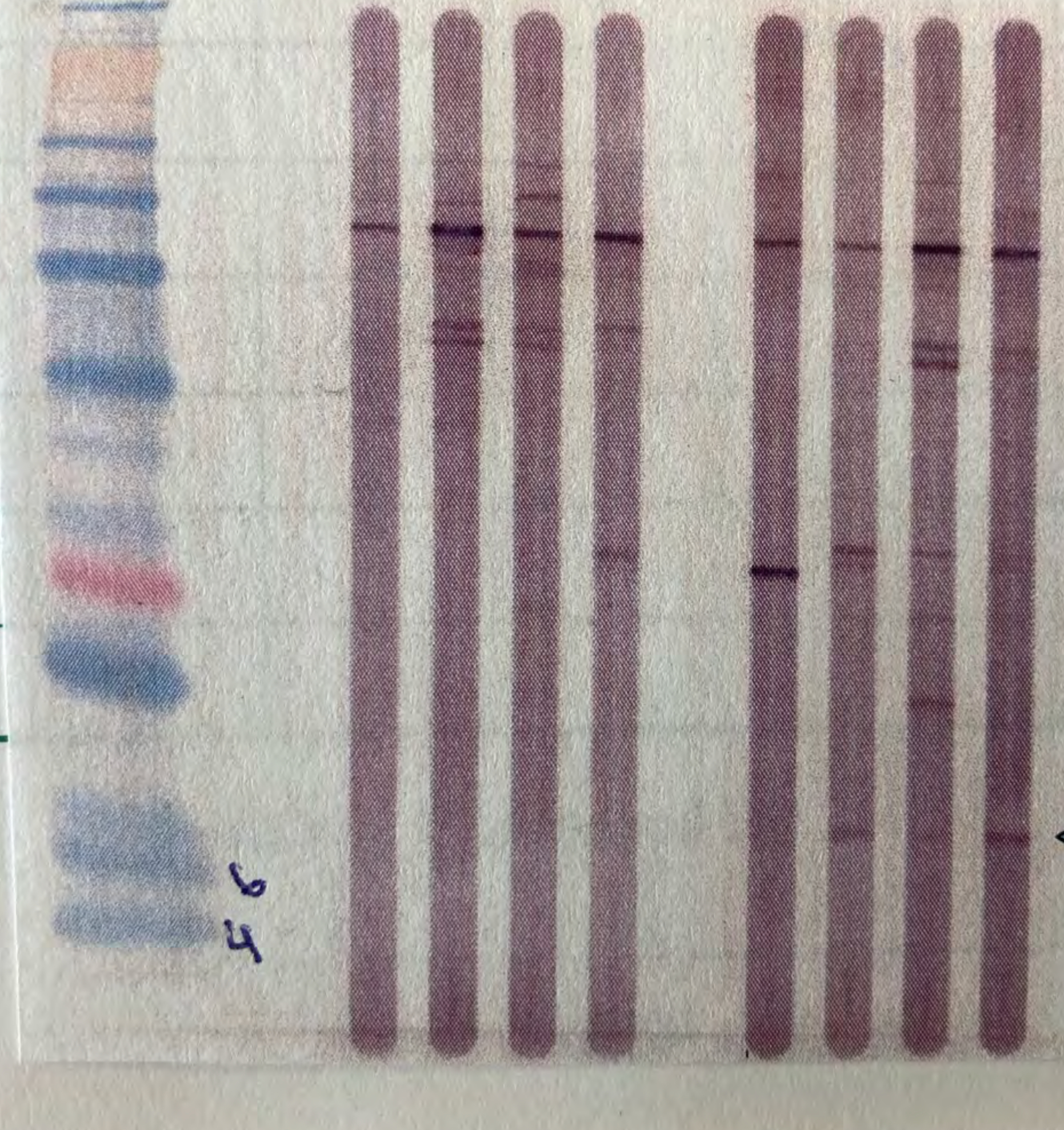

Supplement: S2 File — This is a scanned image of a multiscreen apparatus blot from an experiment unrelated to this article, provided to show the discontinuity in background seen in blots used in this methodology. Once scanned, the image appears flat and the negative lane appears similar to the blank background. (JPG) [file pntd.0012519.s002.jpg]

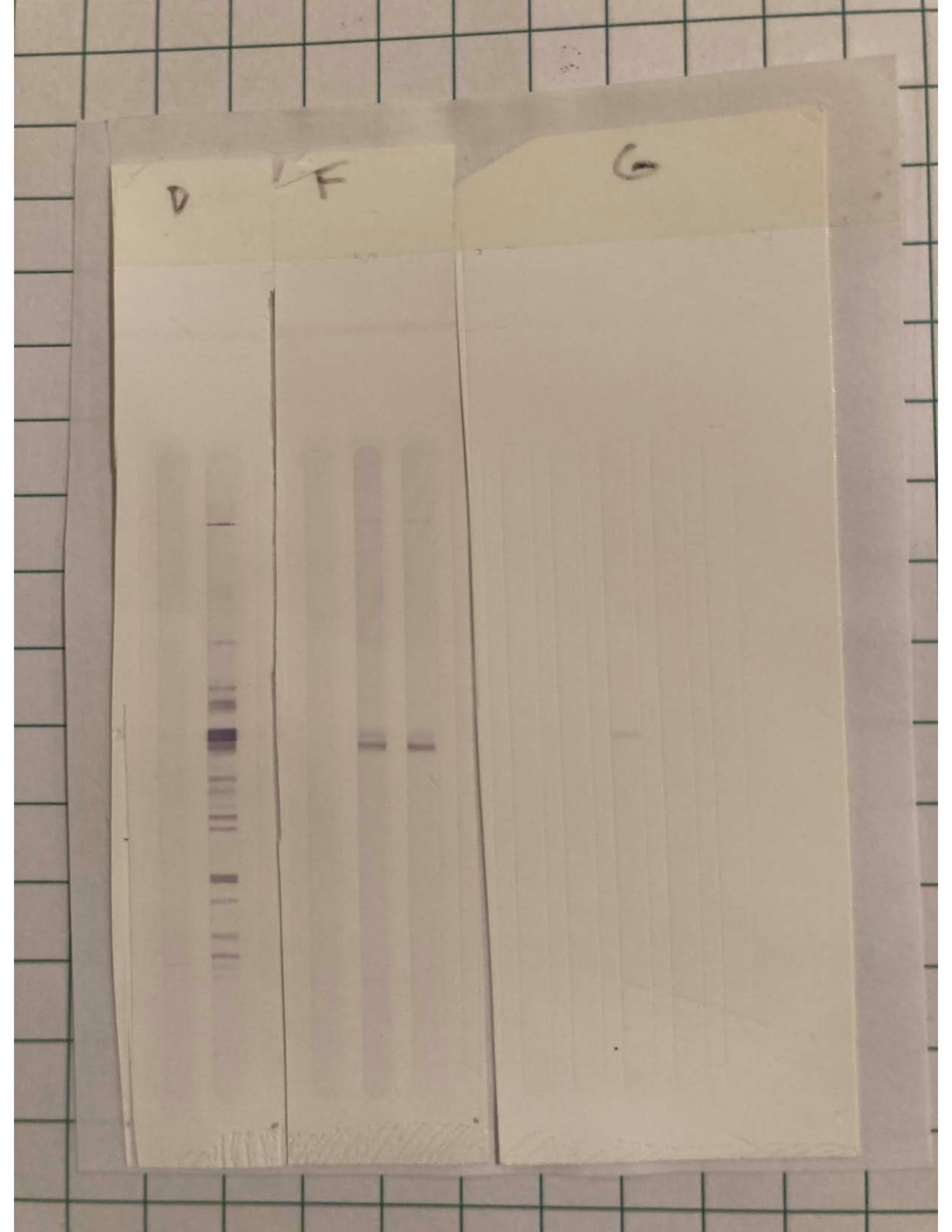

Supplement: S3 File — (JPG) [file pntd.0012519.s003.jpg]

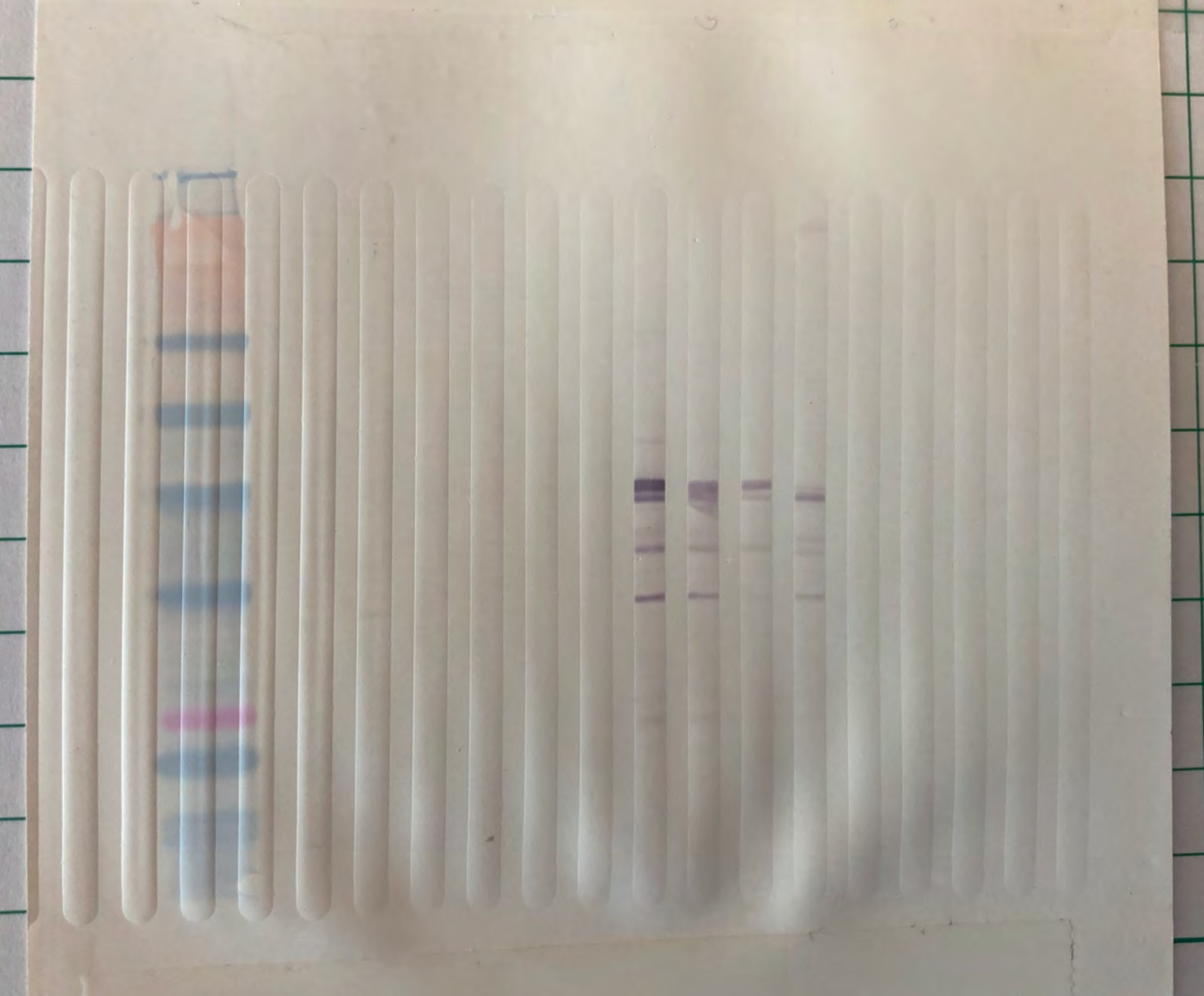

Supplement: S4 File — (JPG) [file pntd.0012519.s004.jpg]

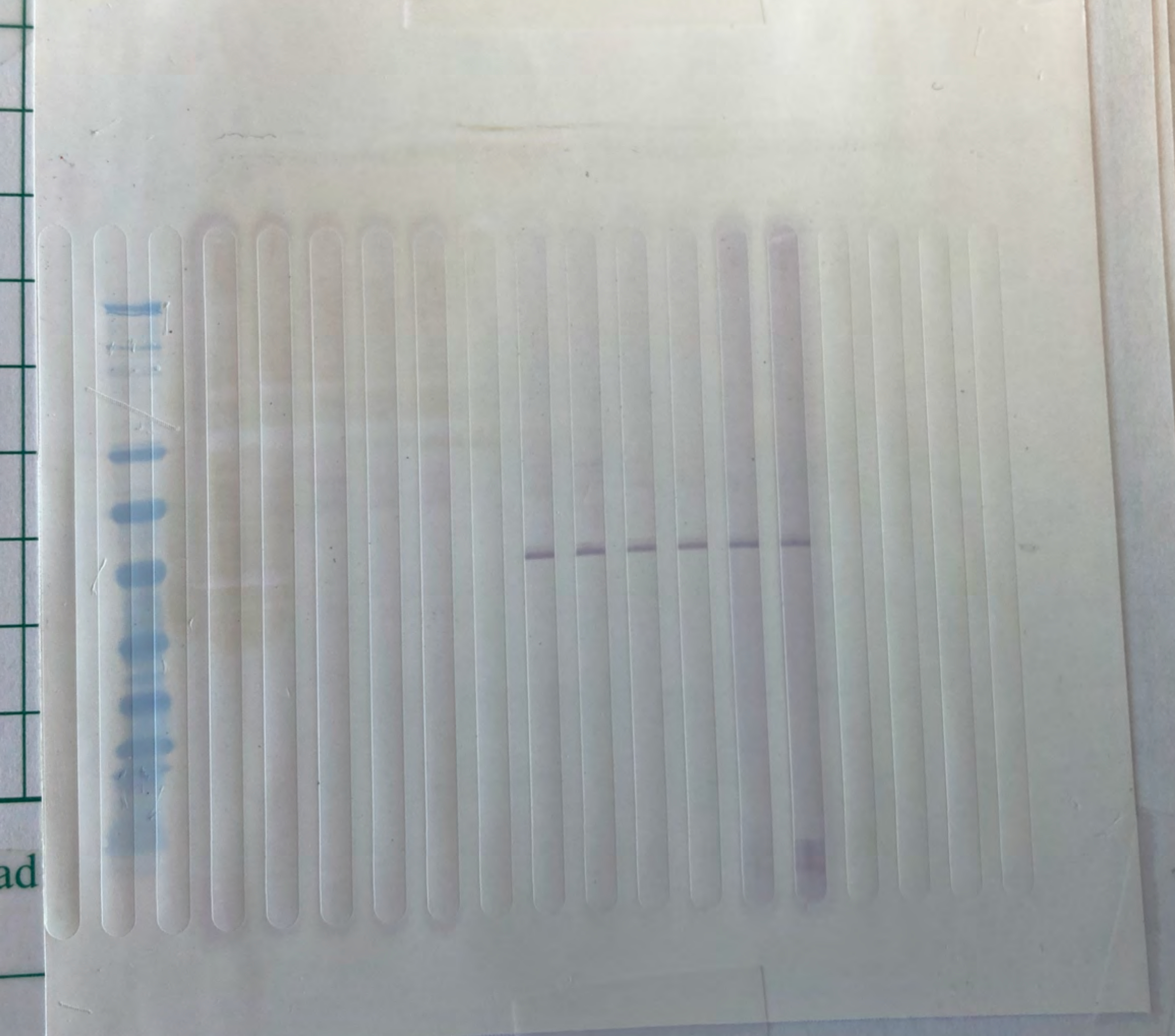

Supplement: S5 File — (JPG) [file pntd.0012519.s005.jpg]

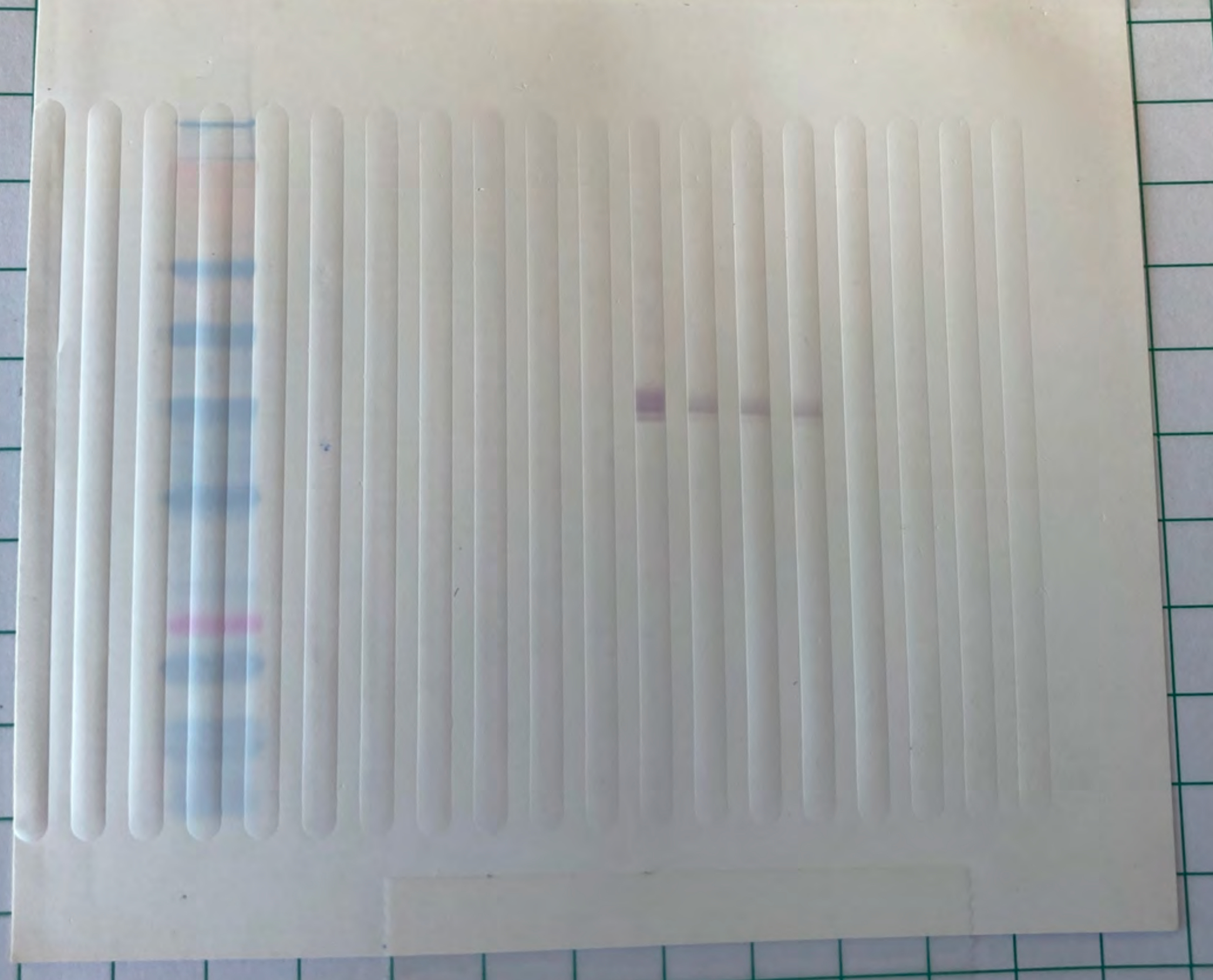

Supplement: S6 File — (JPG) [file pntd.0012519.s006.jpg]

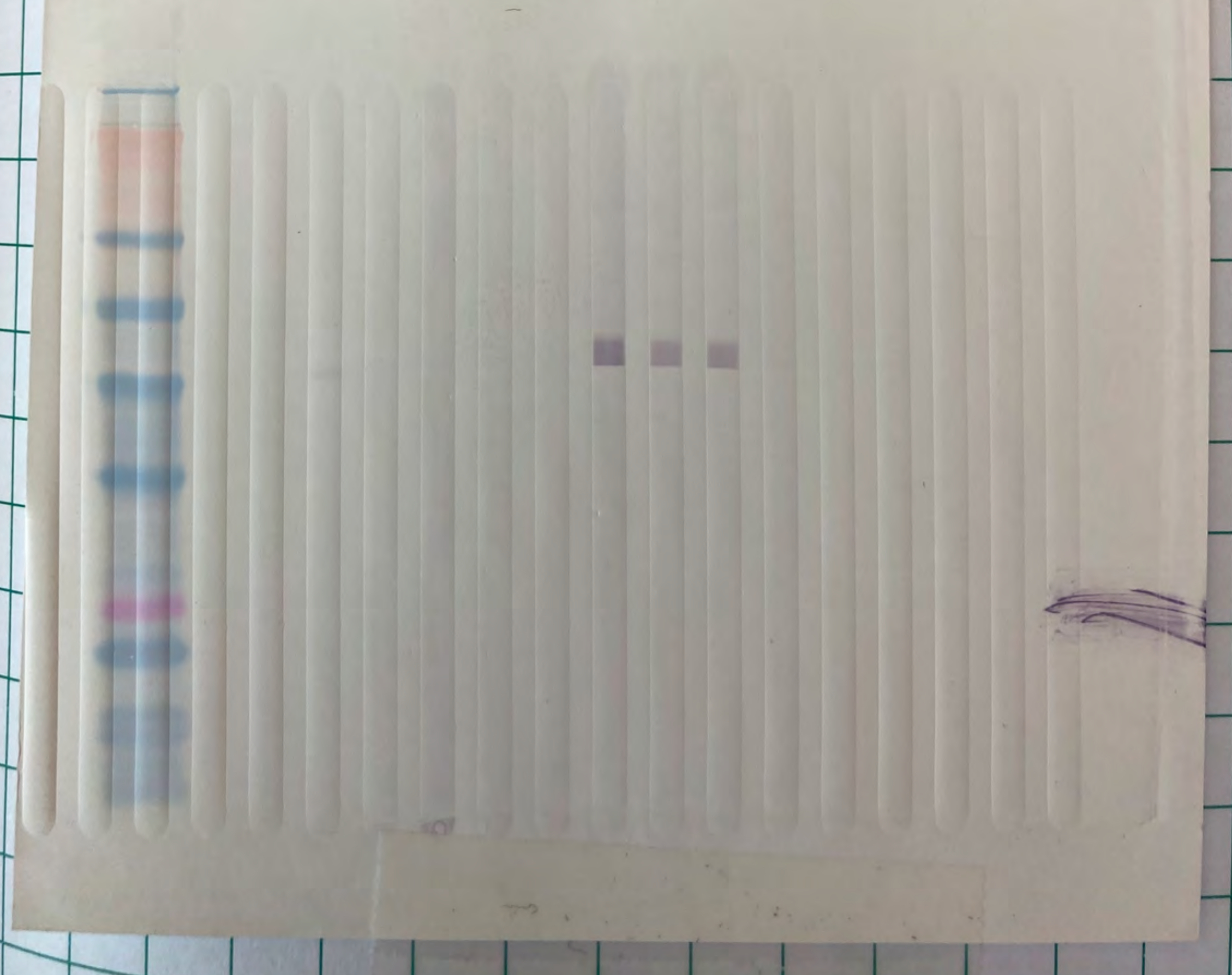

Supplement: S7 File — (JPG) [file pntd.0012519.s007.jpg]
